# Immunosuppressive capacity of mesenchymal stem cells correlates with metabolic activity and can be enhanced by valproic acid

**DOI:** 10.1186/s13287-017-0553-y

**Published:** 2017-04-26

**Authors:** Madeleine C. Killer, Philipp Nold, Katharina Henkenius, Lea Fritz, Tabea Riedlinger, Christina Barckhausen, Miriam Frech, Holger Hackstein, Andreas Neubauer, Cornelia Brendel

**Affiliations:** 1 Zentrum für Tumor- und Immunbiologie (ZTI), Hans-Meerwein-Straße 3, 35043 Marburg, Germany; 2 Biochemisches Institut, Friedrichstraße 24, 35392 Giessen, Germany; 3 Department of Hematology, Oncology and Immunology, Philipps-University Marburg, Baldingerstraße, 35043 Marburg, Germany; 4 Institute for Clinical Immunology and Transfusion Medicine, Justus-Liebig University Giessen, Langhansstraße 7, 35385 Giessen, Germany

**Keywords:** Mesenchymal stem cells, Immunosuppression, T-cell, Cryopreservation, Valproic acid

## Abstract

**Background:**

Mesenchymal stem cells (MSCs) have entered the clinic as an Advanced Therapy Medicinal Product and are currently evaluated in a wide range of studies for tissue regeneration or in autoimmune disorders. Various efforts have been made to standardize and optimize expansion and manufacturing processes, but until now reliable potency assays for the final MSC product are lacking. Because recent findings suggest superior therapeutic efficacy of freshly administered MSCs in comparison with frozen cells, we sought to correlate the T-cell suppressive capacity of MSCs with their metabolic activity.

**Methods:**

Human MSCs were obtained from patients’ bone fragments and were employed in coculture with peripheral blood mononuclear cells (PBMCs) in an allogeneic T-cell proliferation assay to measure immunosuppressive function. Metabolic activity of MSCs was measured in real time in terms of aerobic glycolysis quantified by the extracellular acidification rate and mitochondrial respiration quantified by the oxygen consumption rate.

**Results:**

We show that MSC-induced suppression of T-cell proliferation was highly dependent on individual healthy donors’ lymphocytes. Moreover, coculture with PBMCs increased the glycolytic and respiratory activity of MSCs considerably in a PBMC donor-dependent manner. The twofold to threefold enhancement of cell metabolism was accompanied by higher T-cell suppressive capacities of MSCs. The cryoprotectant dimethyl sulfoxide decreased metabolic and immunosuppressive performances of MSCs while valproic acid (VPA) increased their glycolytic, respiratory and T-cell suppressive capacity.

**Conclusions:**

Functional fitness of MSCs can be determined by measuring metabolic activity and can be enhanced by exposure to VPA. Pretesting the increment of metabolic activity upon interaction of donor MSCs with patient T-cells provides a rational approach for an individualized potency assay prior to MSC therapy.

**Electronic supplementary material:**

The online version of this article (doi:10.1186/s13287-017-0553-y) contains supplementary material, which is available to authorized users.

## Background

Because of their high ex-vivo expansion potential and their immunomodulatory capacity, the therapeutic benefits of mesenchymal stem cells (MSCs) are currently assessed in numerous clinical trials [1, 2]. Promising therapeutic effects have been reported in autoimmune disorders [3], in particular for treatment of multiple sclerosis and graft-versus-host disease (GvHD) [4–7].

While most studies on MSCs as an immunosuppressive cellular therapy product raised new hope for treatment of otherwise refractory patients [5, 8], outcomes of other studies were below expectations [9, 10]. These differences could be explained by the highly varying manufacturing protocols employed for MSC expansion in different studies. Efforts have been made to harmonize and standardize these processes under good manufacturing practice (GMP)-compliant conditions [11, 12]. Moreover, expansion protocols were optimized in order to improve the immunosuppressive performances of MSCs, paving the way for a reliable cellular product that can be administered safely and evaluated in clinical trials [13, 14]. However, an in-vitro potency assay that reliably determines the immunomodulatory capabilities of MSCs is still lacking [15].

Recent studies indicate that freshly administered MSCs may have a superior therapeutic impact compared with frozen cells [16, 17]. In order to elucidate this observation, we aimed to identify the metabolic properties of MSCs in general and under cryopreservative conditions. By conducting simultaneous T-cell proliferation assays and metabolic measurements, we were able to relate the T-cell suppressive capacity of MSCs to their glycolytic and respiratory activity. Interestingly, we found a significant dependency on the peripheral blood mononuclear cell (PBMC) source in these allogeneic MSC–PBMC interaction assays. Furthermore, metabolic activity and also T-cell suppressive capacity of MSCs were consistently reduced by the cryoprotectant dimethyl sulfoxide (DMSO). In contrast, both metabolism and T-cell suppressive capacity were enhanced by exposure of MSCs to valproic acid (VPA).

Our data thus indicate the requirement of a matching MSC–PBMC pair for optimal immunosuppression and provide evidence that metabolic activity is of crucial importance for the immunosuppressive capabilities of MSCs.

## Methods

### Isolation and cultivation of human MSCs

Human MSCs were obtained from bone fragments of patients undergoing hip replacement surgery as approved by the ethics committee at the Philipps-University Marburg (study no. 64/01 and 25/10). MSCs were isolated and cultivated as described previously [11, 14]. Dulbecco’s Modified Eagle Medium (DMEM) containing 1 g/l d-glucose (Gibco by Life Technologies, Carlsbad, CA, USA) supplemented with 1% penicillin/streptomycin (P/S (100×) P11-010; PAA Laboratories GmbH, Pasching, Austria) was supplemented with either 10% fetal calf serum (FCS; Sera Plus; PAN Biotech GmbH, Aidenbach, Germany) or 10% human platelet lysate (HPL). Cells were incubated at 37 °C with 5% CO_2_. MSCs were passaged when they reached ~80% confluence. The immunophenotype and differentiation potential of MSCs were tested as recommended [18] and described previously [11]. MSCs at low passage numbers were frozen in 10% DMSO at –80 °C and stored in liquid nitrogen. Cells were thawed and allowed to equilibrate at least 3 days before being used for further experiments.

### T-cell proliferation assay

The immunomodulatory capacities of human MSCs were investigated using T-cell proliferation assays as described previously [11, 14]. Briefly, MSCs were seeded at densities of 2.5 × 10^4^–1 × 10^5^ cells per well in a 24-well plate. After 24 h of equilibration, PBMCs from healthy donors were isolated from buffy coats via density gradient centrifugation. Subsequently PBMCs were labeled with 1 μM 5,6-carboxyfluorescein succinimidyl ester (CFSE; Molecular Probes, Eugene, OR, USA) and 1 × 10^6^ PBMCs were added per well. T-cell proliferation was induced by addition of CD3 and CD28 antibodies (0.1 μg/ml each; BD Biosciences, Franklin Lakes, NJ, USA). After 5 days of incubation at 37 °C with 5% CO_2_, PBMCs were collected and measured using a BD FACS LSR II with FACS Diva software (BD Biosciences). Results were evaluated using FlowJo™ software (Ashland, OR, USA).

The negative control, in which cells remained unstimulated, was used to define a threshold of the CFSE signal of nonproliferating T-cells. A lower amount of CFSE per cell (in comparison with the negative control) indicates increased proliferation of the respective cells. Percentages of T-cell proliferation after MSC coculture were defined by the CFSE threshold of the respective negative control. All values were calculated as a percentage of the respective positive control. Suppression of T-cell proliferation was then calculated as follows:

$$ 100\%\, \hbox{-}\, \left(\mathrm{T}\mathrm{\hbox{-} cell}\, \mathrm{proliferation}\, \hspace{0.14em}\mathrm{after}\, \mathrm{coculture}\, \left(\%\, \mathrm{of}\, \mathrm{positive}\, \mathrm{control}\right)\right)=\mathrm{T}\mathrm{\hbox{-} cell}\, \mathrm{suppression}\, \left(\%\right). $$

For DMSO pretreatment, MSCs were incubated with 1–5% DMSO for 24 h. VPA pretreatment was carried out with 1 mM VPA for 6 days prior to onset of the assay. DMSO and VPA for pretreatment were removed by media exchange before addition of PBMCs. Addition of 1 mM VPA to the coculture was performed directly after seeding of PBMCs.

### Metabolic analyses

Metabolic studies were performed using the XF96 Extracellular Flux Analyzer (Seahorse Bioscience, North Billerica, MA, USA) which enables the simultaneous real-time measurement of aerobic glycolysis quantified by the extracellular acidification rate (ECAR) and mitochondrial respiration quantified by the oxygen consumption rate (OCR). Human MSCs were seeded at a density of 1 × 10^4^ cells in 80 μl per well in a 96-well plate and measured after 24 h. In the case of measurements of MSCs after coculture with PBMCs, 1 × 10^5^ PBMCs were added to the MSCs after 24 h and the measurement was performed after a further 24 h. One hour before the measurement, cells were washed and culture media were replaced with low-glucose media. DMSO or VPA pretreatment of MSCs was performed as already described. Six to eight replicates were performed for each condition within each measurement. Basal measurements of ECAR and OCR as well as measurements after addition of glucose (final concentration 10 mM; Sigma Aldrich, St. Louis, MO, USA), oligomycin (final concentration 5 μM; Sigma Aldrich) and 2-deoxy-d-glucose (2DG, final concentration 100 mM; Seahorse Bioscience) were performed as described in the XF Glycolysis Stress Test Kit User Manual (Seahorse Bioscience). All media and solutions were prepared and applied as recommended by the manufacturer. Oligomycin was dissolved to 5 mM in DMSO.

To exclude tampering of metabolic data by varying cell numbers, ECAR and OCR values were normalized to the protein content of the respective well. Protein concentration was determined with the Pierce BCA Protein Assay (Thermo Scientific, Waltham, MA, USA). Absorbance at 540 nm was measured with a microplate absorbance reader provided with Magellan data analysis software (Tecan sunrise; Tecan, Männedorf, Switzerland). A bovine serum albumin (BSA) standard curve was included in each measurement.

### Statistical analyses

All statistical data analyses were performed using GraphPad Prism 5 software (San Diego, CA, USA). Error bars indicate mean ± SEM. For analyses of correlation the Pearson’s *r* value was determined. Group comparison was calculated employing the Student’s *t* test or one-way ANOVA with Bonferroni correction.

## Results

### Variation of MSC-induced T-cell suppression of lymphocytes from healthy individuals

**Fig. 1:**
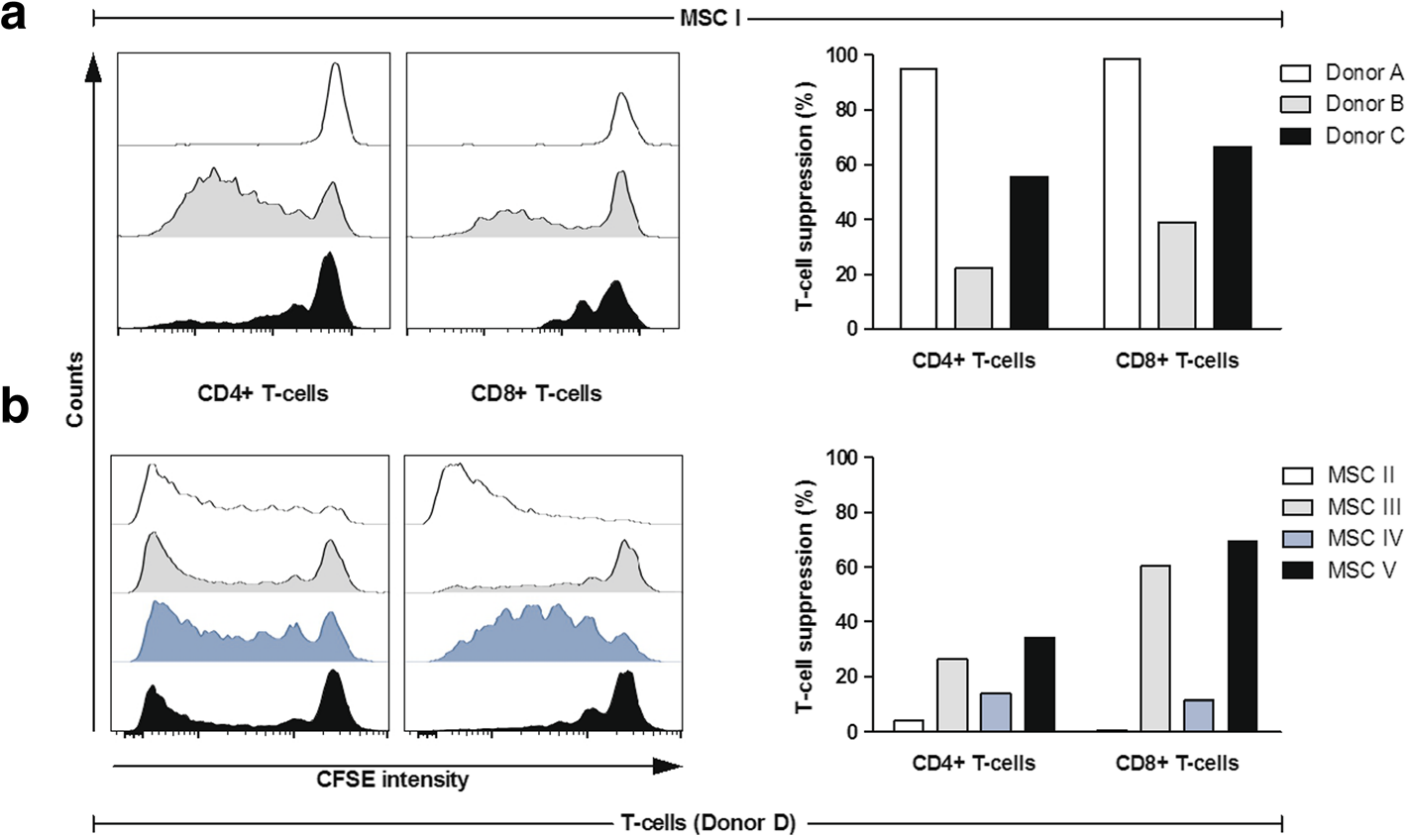
MSC-induced T-cell suppression depends on MSC and T-cell donors. Proliferation of CFSE-labeled CD4^+^ as well as CD8^+^ T-cell subpopulations was induced with CD3/28 antibodies and the CFSE intensity was measured via flow cytometry. The exemplary result of one representative experiment is shown (*n* = 1). **a** PBMCs from three different donors were cocultured with MSCs from one batch. T-cells responded differently to MSC-mediated suppression. **b** PBMCs from one donor were cocultured with MSCs from four different batches. Extent of T-cell suppression varied distinctly between different MSC batches. *CFSE* carboxyfluorescein succinimidyl ester, *MSC* mesenchymal stem cell

In order to determine the capability of MSCs to suppress the proliferation of CD3/CD28 antibody-stimulated T-cells, every MSC batch was tested in a coculture assay with PBMCs from three different healthy donors in parallel. Broad testing of 29 MSC and 65 PBMC batches revealed that immunosuppression was highly variable between different PBMC as well as MSC donor samples. MSCs accomplished high suppression of T-cell proliferation in some PBMC samples but were far less successful in others, as depicted in Fig. 1a and Additional file 1: Figure S1A, B for one representative MSC batch, respectively. The suppression ranged from 22 to 98%. Variations of T-cell suppressive capacity were also observed in different MSC batches tested with the same PBMC donor. Figure 1b and Additional file 1: Figure S1C, D illustrate results for a single donor PBMC and four MSC batches, respectively. Here, the suppression was between 0.5 and 69%. Monitoring cell proliferation of CD4^+^ and CD8^+^ cells gave similar results. The highest variation, however, was observed when CD8^+^ T-cell proliferation was determined after coculture with MSCs from different donors. Thus, potency for in-vitro immunosuppressive activity of MSCs does not simply rely on specific MSC batch performances but highly depends on the predisposition of lymphocytes from different donors. A correlation between donor age and T-cell suppression was not observed (Additional file 1: Figure S2).

### Interaction with PBMCs enhances MSC metabolism

Furthermore, we sought to identify factors that determine the donor dependency of PBMC and MSC interactions in coculture. As shown previously, senescence of MSCs is correlated with poor T-cell suppressive capacity [14]. Because senescent MSCs exhibit low metabolic activity [19], we sought to analyze the metabolism of MSCs and PBMCs in coculture in terms of the ECAR (i.e., cellular lactate extrusion as a surrogate of glycolysis) and the OCR (as an indicator of cellular respiration).

**Fig. 2:**
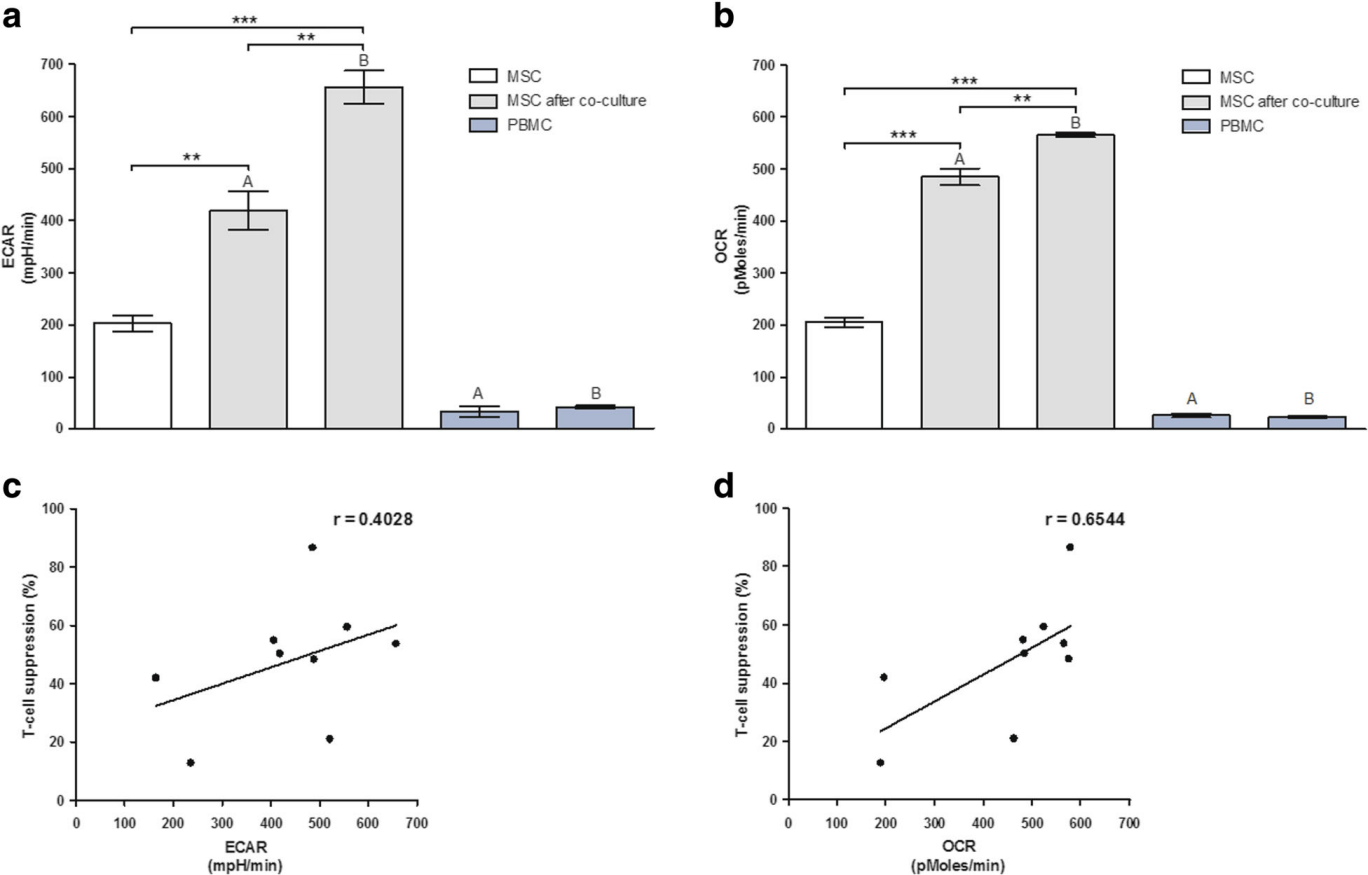
Immunosuppressive capacity of MSCs correlates with glycolytic and respiratory activity. MSCs were cocultured with PBMCs from different donors and subjected to T-cell proliferation assays as well as metabolic measurements simultaneously. PBMC-dependent increase of **a** ECAR and **b** OCR of MSCs after 24 h of coculture with two different PBMC donors (donor A and donor B) in comparison with MSCs in monoculture (*n* = 1 in sixfold repetition). ECAR and OCR of PBMCs in monoculture were measured as a control. **c** ECAR and **d** OCR of MSCs correlate with their T-cell suppressive capacity (*n* = 9). *ECAR* extracellular acidification rate, *MSC* mesenchymal stem cell, *OCR* oxygen consumption rate, *PBMC* peripheral blood mononuclear cell, r Pearson’s *r* value. *p ≤ 0.05, **p ≤ 0.01, ***p ≤ 0.001

Coculture of MSCs with proliferating PBMCs led to a twofold to threefold enhancement of both MSC ECAR and MSC OCR compared with the monoculture, as illustrated in Fig. 2a, b. This shift did not rely on PBMC metabolic activity, as verified by control measurements of PBMCs in monoculture. Measurements of PBMCs taken from the cocultures showed no detectable levels of metabolic activity (data not shown). The increased rate of ECAR and OCR in MSCs differed between PBMC donor samples. Moreover, we found a correlation between the extent of MSC ECAR and OCR activity and their ability to suppress T-cell proliferation as shown in Fig. 2c, d. In summary, metabolism of MSCs is significantly increased upon PBMC coculture in a donor-dependent manner and metabolic activity correlates with their T-cell suppressive ability.

### Dimethyl sulfoxide impairs the immunosuppressive and metabolic activity of human MSCs

**Fig. 3:**
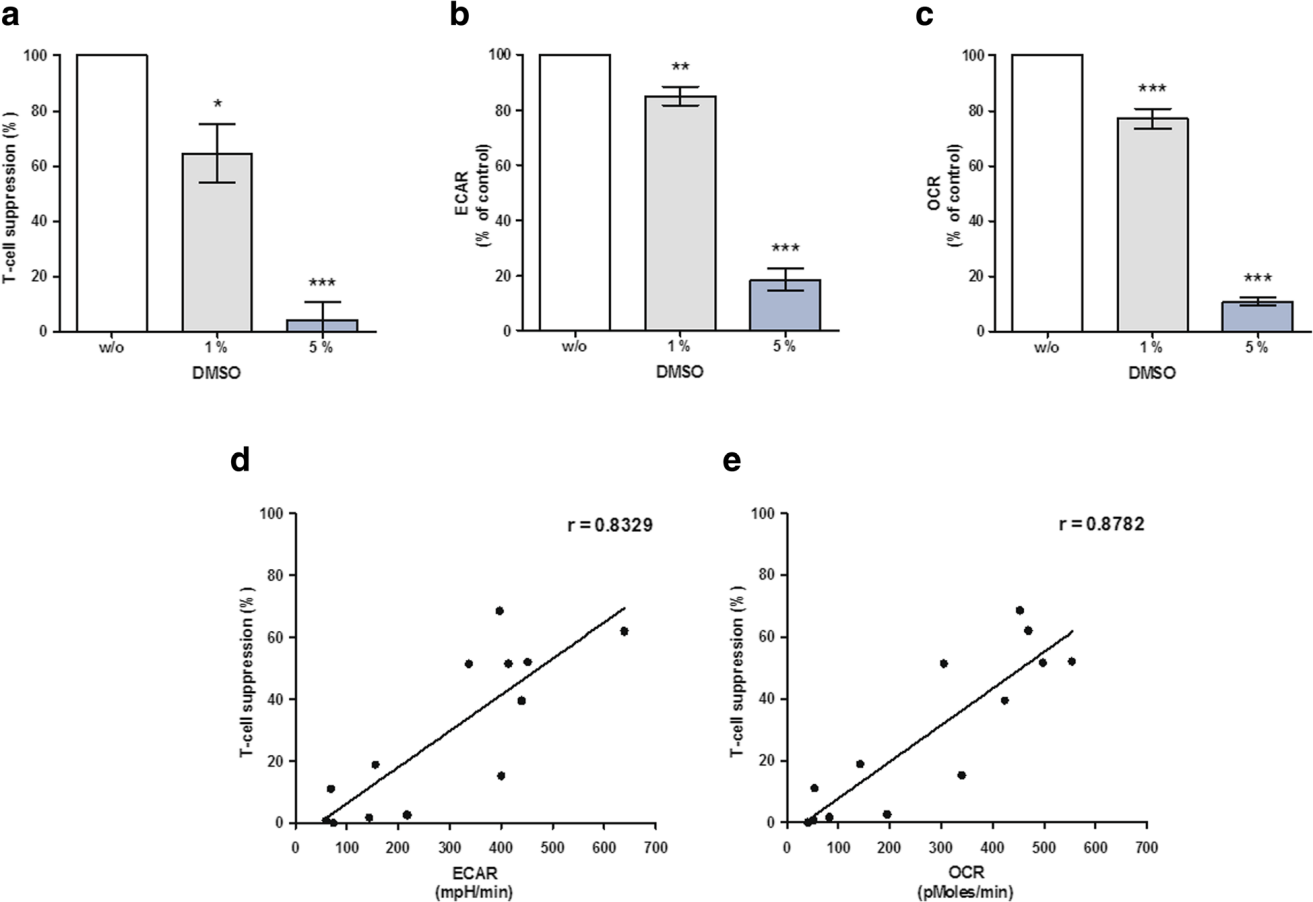
Dimethyl sulfoxide impairs the T-cell suppressive capacity, glycolysis and cellular respiration of MSCs. MSCs of various batches were pretreated with 1 or 5% DMSO for 24 h, cocultured with PBMCs from different donors and subjected to T-cell proliferation assays. Metabolic measurements were performed simultaneously. **a** DMSO impairs MSC-mediated CD4^+^ T-cell inhibition in a dose-dependent manner (*n* ≥ 4). Data were normalized to untreated MSCs. DMSO diminishes **b** ECAR and **c** OCR of MSCs dose dependently (*n* ≥ 4). **d** ECAR and **e** OCR of DMSO-treated MSCs correlate with their suppressive capacity toward CD4^+^ T-cells (*n* = 13). *DMSO* dimethyl sulfoxide, *ECAR* extracellular acidification rate, *OCR* oxygen consumption rate, r Pearson’s *r* value. *p ≤ 0.05, **p ≤ 0.01, ***p ≤ 0.001

We reported previously that cryopreservation can impair the immunosuppressive function of MSCs [14] and others have suggested a superior efficacy of fresh versus frozen MSCs for patient treatment [20]. Therefore we sought to determine the impact of the cryoprotectant DMSO on the metabolism and on the T-cell suppressive abilities of human MSCs. Pretreatment with DMSO decreased the T-cell suppressive capacity of MSCs in a dose-dependent manner (Fig. 3a). Likewise, DMSO pretreatment attenuated the ECAR and OCR of MSCs in monoculture (Additional file 1: Figure S3) and in PBMC coculture (Fig. 3b, c). Again, parallel monitoring of immunosuppression and ECAR or OCR of MSCs after DMSO pretreatment demonstrated a clear correlation (Fig. 3d, e), confirming the strong impact of DMSO on MSC functioning. These observations suggest that freezing MSCs with DMSO impairs MSC metabolism in a similar way. Previously frozen MSCs were therefore subjected to metabolic measurements directly after thawing and after equilibration times of 24–72 h. The ECAR and OCR of MSCs were low directly after thawing but recovered during equilibration (Additional file 1: Figure S4). In summary, DMSO impairs the immunomodulatory as well as metabolic activity of human MSCs. Moreover, the previously observed correlation between metabolism and T-cell suppressive capacities was confirmed for MSCs exposed to DMSO.

### Valproic acid enhances the immunosuppressive and metabolic activity of human MSCs

**Fig. 4:**
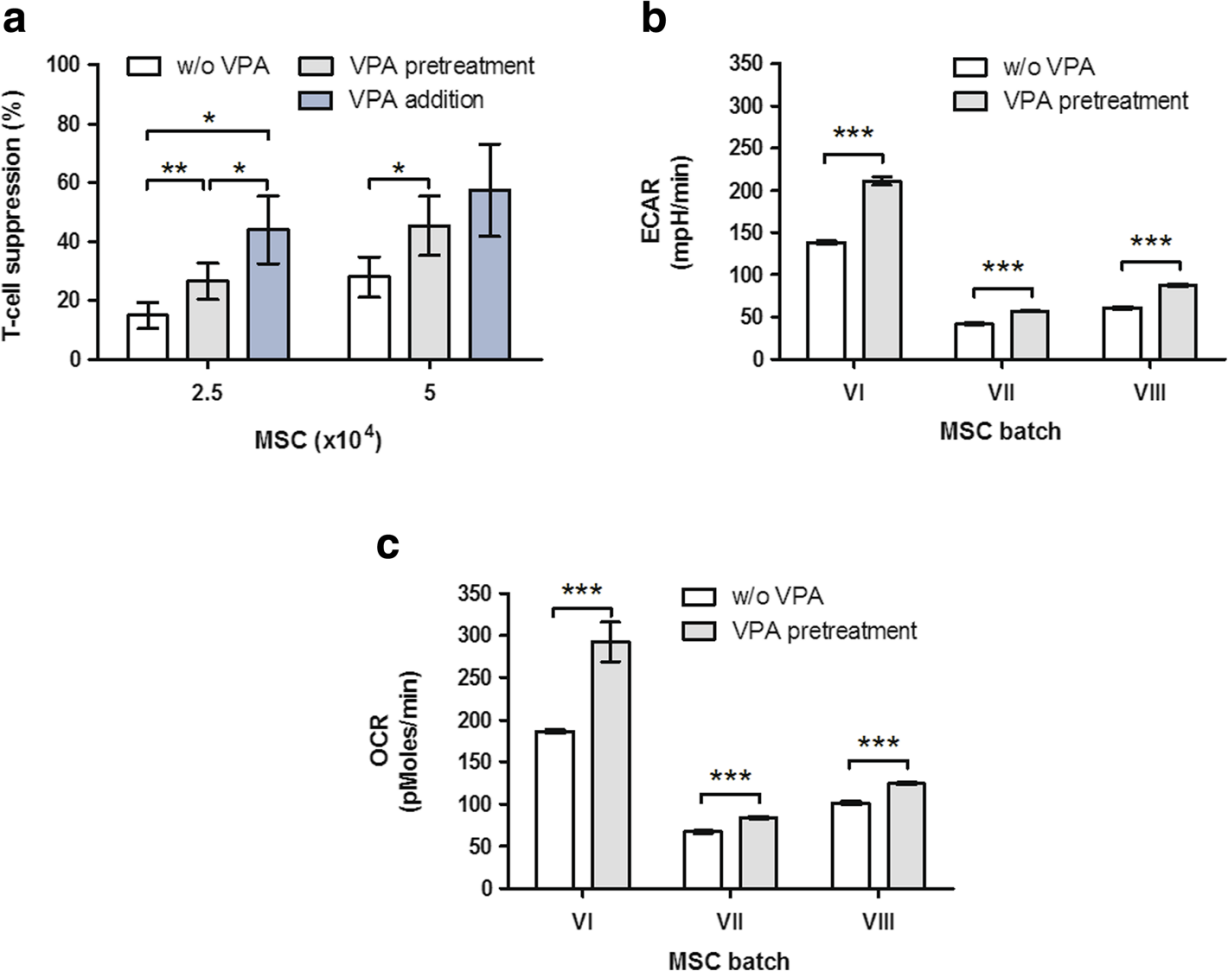
Valproic acid enhances the immunosuppressive activity as well as the glycolysis and cellular respiration of MSCs. MSCs of various batches were pretreated with 1 mM VPA for 6 days and were either subjected to T-cell proliferation assays with PBMCs or to metabolic measurements in monoculture. **a** VPA increases the MSC-mediated T-cell inhibition. Suppressive capacity toward CD4^+^ T-cells is shown for MSCs seeded at densities of 2.5 × 10^4^ (*n* = 7) and 5 × 10^4^ (*n* = 3) cells. VPA (1 mM) was used either for pretreatment of MSCs before onset of the assay or directly in coculture of PBMCs with untreated MSCs. Data were normalized to T-cell proliferation without the presence of MSCs. VPA-dependent increase of MSC **b** ECAR and **c** OCR after 6 days of VPA pretreatment (*n* = 1 in eightfold repetition). *ECAR* extracellular acidification rate, *MSC* mesenchymal stem cells, *OCR* oxygen consumption rate, *VPA* valproic acid. *p ≤ 0.05, **p ≤ 0.01, ***p ≤ 0.001

Treatment of human MSCs with the histone deacetylase (HDAC) inhibitor VPA was shown to enhance cell motility, viability under oxidative stress and the secretion of trophic factors [21]. Therefore, we sought to determine the effects of VPA on metabolism and the T-cell suppressive capacity of MSCs. Pretreatment of MSCs with VPA resulted in a stronger inhibition of T-cell proliferation compared with the inhibition exerted by untreated MSCs (Fig. 4a). However, this outcome was only detectable if T-cells from the respective PBMC donor were generally susceptible to MSC-mediated inhibition (see also Fig. 1), which occurred in six out of nine PBMC donors who were analyzed in this context. In general, the T-cell suppressive effect was MSC dose dependent as shown previously [11]. The supportive effect of VPA on MSC-mediated T-cell inhibition was even stronger if VPA was added directly to the MSC–PBMC coculture (Fig. 4a). Besides, a direct suppressive effect of VPA on T-cell proliferation was observed. T-cell suppression by VPA in the absence of MSCs ranged from 7 to 77%, with a mean suppression of 31% (*n* = 12). Furthermore, measurements of metabolic activity of three different MSC batches revealed that ECAR as well as OCR of all analyzed batches were enhanced after VPA pretreatment (Fig. 4b, c). An essential negative effect on viability of PBMCs was not observed (Additional file 1: Figure S5). In general, metabolic activity varied between individual MSC batches. Thus, VPA attenuates T-cell proliferation directly and enhances metabolic and immunosuppressive activity of MSCs.

## Discussion

We and others have shown previously that MSCs inhibit T-cell proliferation in a dose-dependent manner [11, 22]. We show a high variation of T-cell suppressive capacity of various MSC batches on the same PBMC donor samples which is consistent with recently published data [23]. Interestingly, the response of different PBMC donor samples to the immunosuppressive capability of the same MSC batch also revealed a substantial variation. Because of similar observations, Ketterl et al. [24] used pooled leukocyte samples in order to reduce inconsistencies in immune responses of individual T-cell samples. However, failure of T-cell suppression as an inherent disability of lymphocytes from healthy individuals has not been described systematically. It has been suggested that T-cells from patients with autoimmune disorders exhibit less reactivity to MSC-induced suppression of proliferation compared with healthy individuals and that the inhibitory function is mediated through monocytes [25]. We observed that unresponsiveness of donor T-cells to MSC-mediated suppression occurred even amongst healthy individuals, while the same MSC batches exhibited high immunosuppressive action toward T-cells from other donors. Additionally, MSCs from different individuals act differently at the level of direct interaction with PBMCs. We further showed that different PBMC batches enhance MSC ECAR and OCR to variable degrees. MSCs that are highly capable of suppressing T-cell proliferation react with a significant enhancement of metabolism in response to PBMC coculture, suggesting a dependency of T-cell suppressive capacity of MSCs on metabolic activity. Accordingly, a linear correlation of metabolic activity and T-cell suppressive capacity of MSCs was observed. In line with the observation of low glycolytic activity in senescent MSCs [26], we could previously link diminished T-cell suppression of MSCs with senescence [14].

Advanced Therapy Medicinal Products (ATMPs) are often frozen employing 5–10% DMSO [27]. We therefore corroborated our hypothesis by demonstrating a DMSO dose-dependent impairment of metabolic activity and immunosuppressive function of MSCs. In contrast, we showed that pretreatment of MSCs with VPA induced metabolic activity. Furthermore, we found that VPA directly reduced T-cell proliferation. Anti-proliferative and apoptosis-inducing effects of VPA on T-cells have been described in-vitro and in-vivo [28]. Beyond this T-cell modulating effect, MSCs pretreated with VPA displayed a superior function to suppress T-cell proliferation compared with untreated MSCs. When adding VPA directly to the MSC–PBMC coculture, we observed a further increase of MSC-mediated T-cell suppression. Because HDAC inhibitors exhibit an immunosuppressing effect when applied for treatment of GvHD [29], our data support the notion that combined application of MSCs plus VPA could be a very active treatment regimen for GvHD. In line with this, VPA was shown to increase frequency and function of regulatory T-cells (T-regs) in a mouse model of immune-mediated arthritis which correlated with reduced incidence and severity of the disease [30]. In-vitro immunosuppression via increased amounts of T-regs is a mechanism also described for MSCs [31].

In search of a suitable potency assay for MSCs, T-cell proliferation assays with pooled donor T-cells have been proposed [32]. In contrast, our data indicate that measurement of individual patient T-cell response to MSCs could be more relevant in assessment of efficiency of MSC therapy. Based on the correlation of MSC metabolism with their immunosuppressive potential, we suggest that MSC functionality can be predicted via metabolic measurements such as lactate acid production or oxygen consumption after patient PBMC and donor MSC coculture. Taken together, our findings point to a feasible and informative potency assay that considers interindividual variations in the interaction of MSCs with patient immune effector cells.

## Conclusion

Our data pave the way for an individualized potency assay for donor MSC and recipient T-cell interaction based on metabolic measurements. Enhancement of T-cell suppressive function through VPA supports the notion that the immunosuppressive activity of MSCs can be further enhanced in-vitro and potentially in-vivo. Whether metabolic parameters could be useful to predict the efficacy of MSC therapy in-vivo needs to be determined in prospective clinical trials.

## Additional files

Additional file 1: Figure S1. Showing that T-cell suppression induced by MSCs is heterogeneous, **Figure S2.** Showing that PBMC predisposition to MSC-mediated suppression does not correlate with donor age, **Figure S3.** Showing that DMSO pretreatment attenuates the ECAR and OCR of MSCs, **Figure S4.** Showing that freezing with DMSO attenuates MSC metabolism and **Figure S5.** Showing the influence of VPA and DMSO treatment on PBMC survival. (DOCX 2025 kb)

## Abbreviations

2DG: 2-Deoxy-d-glucose
ATMP: Advanced Therapy Medicinal Product
CFSE: 5,6-Carboxyfluorescein succinimidyl ester
DMSO: Dimethyl sulfoxide
ECAR: Extracellular acidification rate
GMP: Good manufacturing practice
GvHD: Graft-versus-host disease
HDAC: Histone deacetylase
MSC: Mesenchymal stem cell
OCR: Oxygen consumption rate
PBMC: Peripheral blood mononuclear cell
T-reg: Regulatory T-cell
VPA: Valproic acid
