# Supplementary material for: Immunosuppressive capacity of mesenchymal stem cells correlates with metabolic activity and can be enhanced by valproic acid
Source: Stem Cell Res Ther. 2017 Apr 26;8:100. doi: 10.1186/s13287-017-0553-y (PMC5406996; doi:10.1186/s13287-017-0553-y)
Supplement: Supplementary file 1 — Showing that T-cell suppression induced by MSCs is heterogeneous, Figure S2. Showing that PBMC predisposition to MSC-mediated suppression does not correlate with donor age, Figure S3. Showing that DMSO pretreatment attenuates the ECAR and OCR of MSCs, Figure S4. Showing that freezing with DMSO attenuates MSC metabolism and Figure S5. Showing the influence of VPA and DMSO treatment on PBMC survival. (DOCX 2025 kb) [file 13287_2017_553_MOESM1_ESM.docx]

**Supplementary results**


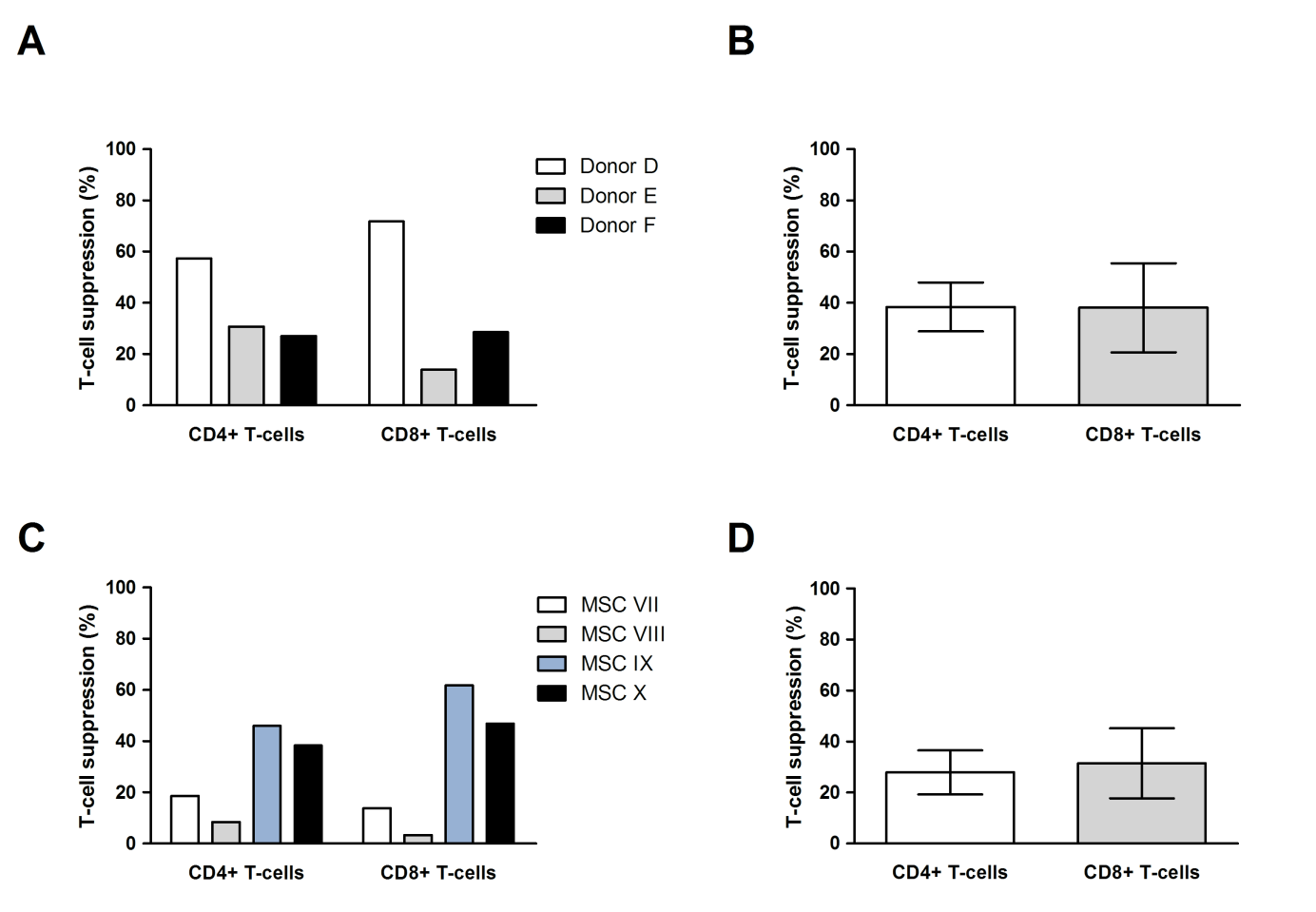


Figure 1S: T-cell suppression induced by MSCs is heterogeneous.

Proliferation of CFSE labeled CD4^+^ as well as CD8^+^ T-cell subpopulations was induced with CD3/28 antibodies and CFSE intensity was measured via flow cytometry. (A) PBMC from 3 different donors showed a heterogeneous response to suppression mediated by one single MSC-batch (n = 1). (B) Suppression values of PBMCs from (A) merged together in one bar graph per T‑cell subpopulation. (C) MSCs from 4 different donors were co-cultured with one PBMC batch (n = 1). Variation in T-cell suppression of different MSCs is shown. (D) Suppression values of PBMCs from (C) merged together in one bar graph per T‑cell subpopulation. Abbreviations: MSCs, mesenchymal stem cells.


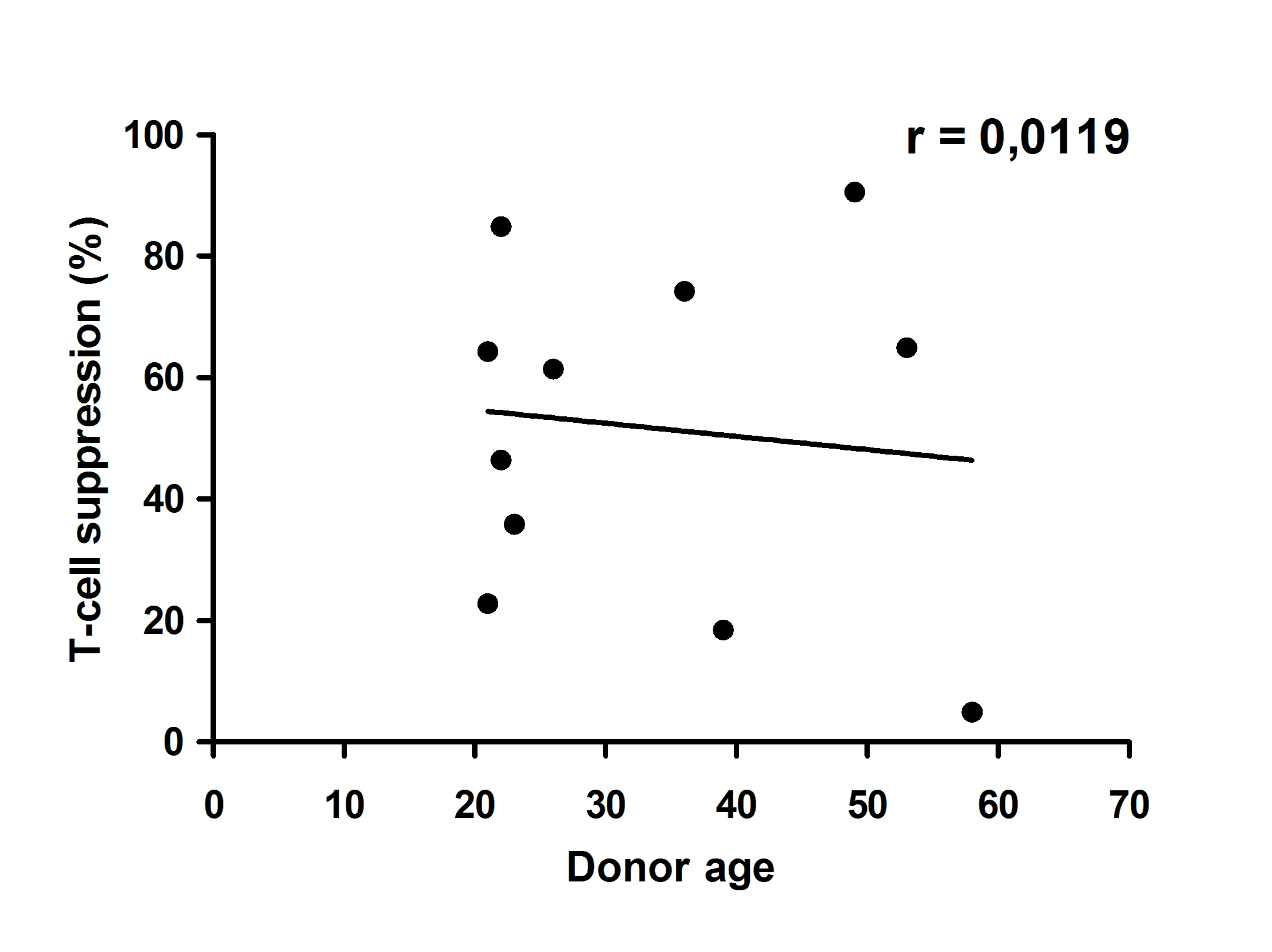


Figure 2S: PBMCs’ predisposition to MSC-mediated suppression does not correlate with donor age.

MSC-mediated T-cell suppression from co-culture experiments was plotted against the age of the corresponding PBMC donors. No correlation was found in the tested cohort (n = 11; r = Pearson’s *r* value).


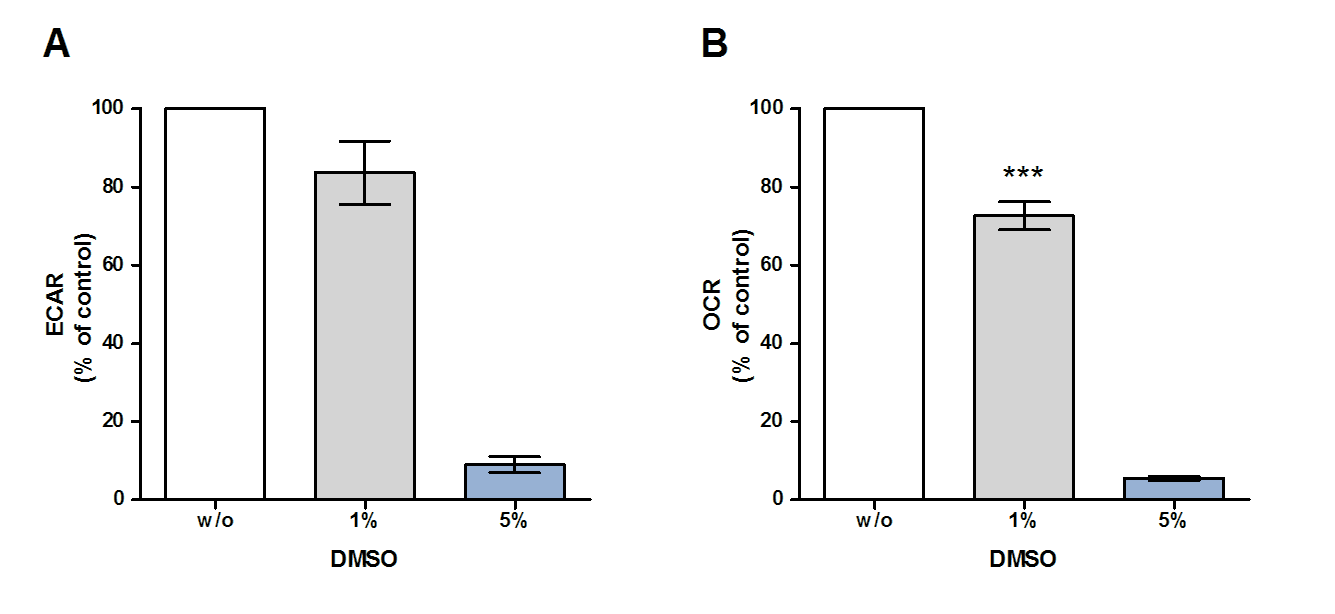


**Figure 3S: DMSO pretreatment attenuates the ECAR and OCR of MSCs.**

MSCs of various batches were pretreated with 1 or 5 % DMSO for 24 h and subjected to metabolic measurements. DMSO impairs **(A)** ECAR and **(B)** OCR of MSCs in a dose-dependent manner (n ≥ 2). Data were normalized to untreated MSCs. Abbreviations: DMSO, dimethyl sulfoxide; ECAR, extracellular acidification rate; OCR, oxygen consumption rate.


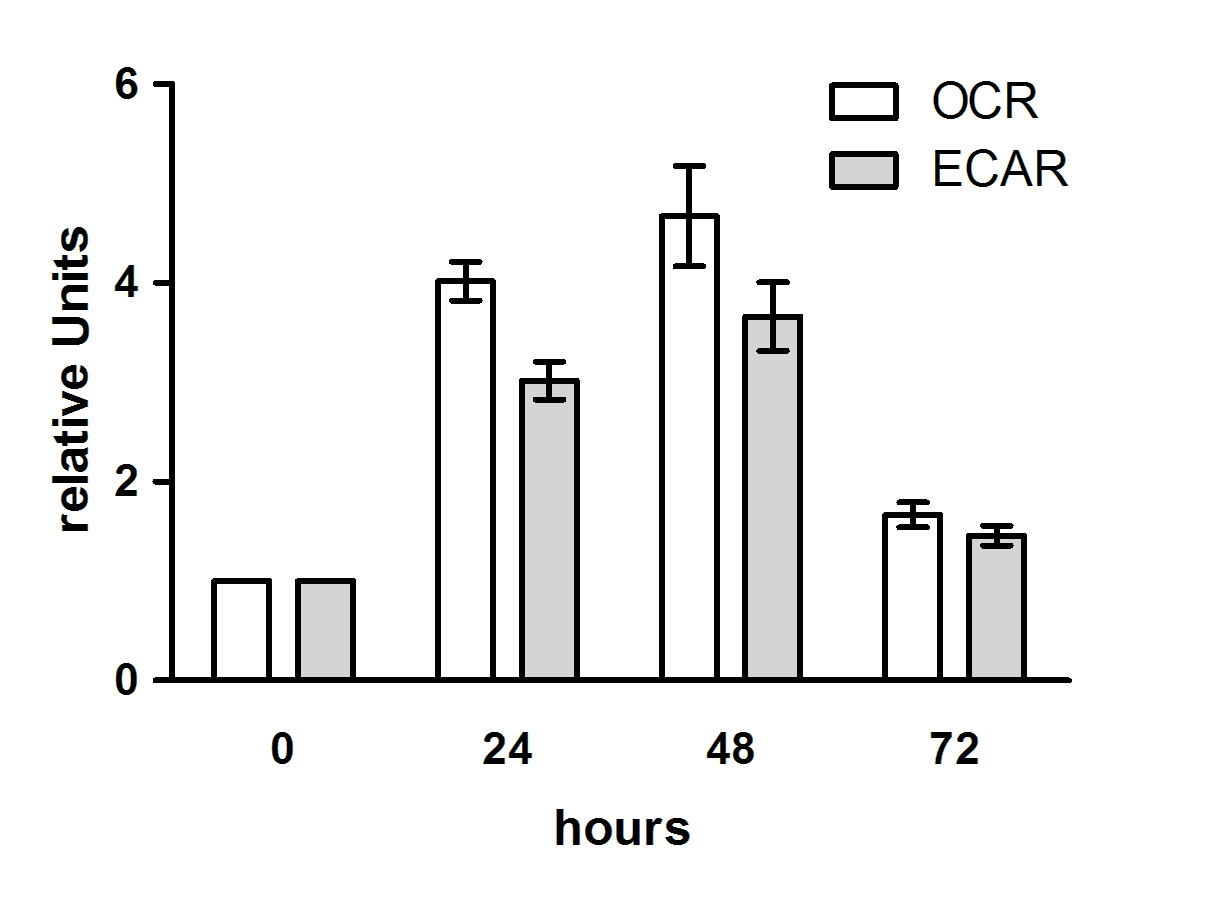


**Figure 4S: Freezing with DMSO attenuates MSCs’ metabolism.**

MSCs were frozen in 10 % DMSO after isolation and stored in liquid nitrogen. Before onset of the experiment, MSCs from different batches were thawed and either subjected to metabolic measurements directly after thawing (0 hours) or were allowed to equilibrate for 24 to 72 hours before measurement. ECAR as well as OCR of MSCs were low directly after thawing but recovered during equilibration. Abbreviations: ECAR, extracellular acidification rate; OCR, oxygen consumption rate.


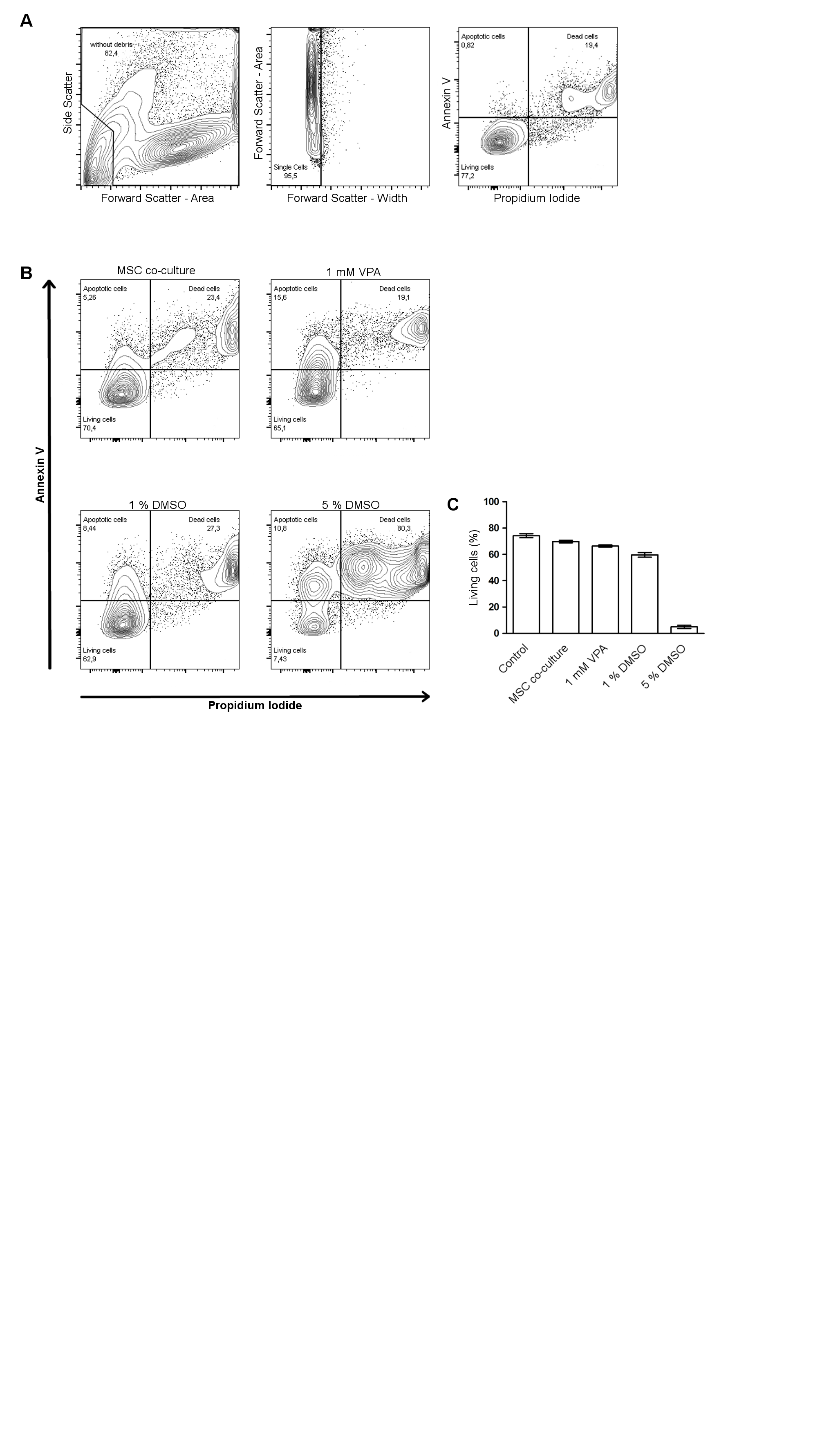


**Figure 5S: Influence of VPA and DMSO treatment on PBMC survival**

PBMCs were either co-cultured with MSCs or treated with VPA or DMSO at the indicated concentrations for 5 days and apoptosis was measured via flow cytometry. **(A)** Gating strategy for the detection of apoptotic cells as illustrated with the untreated control: Cell debris and doublets were excluded via light scatter parameters. Annexin V and propidium iodide staining were used to distinguish between living, apoptotic and dead cells. **(B)** Comparison of apoptosis of PBMCs after the indicated treatments. One exemplary PBMC donor is shown. **(C)** Graphical presentation of surviving PBMCs after the indicated treatments (n = 3). Abbreviations: DMSO, dimethyl sulfoxide; MSCs, mesenchymal stem cells; VPA, valproic acid.
